# Antifungal Activity and Mechanism of Action of the Co(III) Coordination Complexes With Diamine Chelate Ligands Against Reference and Clinical Strains of *Candida* spp.

**DOI:** 10.3389/fmicb.2018.01594

**Published:** 2018-07-18

**Authors:** Katarzyna Turecka, Agnieszka Chylewska, Anna Kawiak, Krzysztof F. Waleron

**Affiliations:** ^1^Department of Pharmaceutical Microbiology, Faculty of Pharmacy, Medical University of Gdańsk, Gdańsk, Poland; ^2^Department of Bioinorganic Chemistry, Faculty of Chemistry, University of Gdańsk, Gdańsk, Poland; ^3^Department of Biotechnology, Intercollegiate Faculty of Biotechnology, University of Gdańsk and Medical University of Gdańsk, Gdańsk, Poland

**Keywords:** *Candida* spp., Co(III) coordination complexes, antifungal activity, microbroth dilution method, MIC, synergy, cytotoxicity

## Abstract

Although many antifungal agents are available in clinical treatment, increasing resistance of fungi, especially *Candida* species, to the available drugs requires the development of new safe and non-toxic compounds with novel modes of action as effective treatment against resistant microorganisms. Cobalt complexes are very interesting and attractive as potential candidates with antimicrobial activity. Their therapeutic uses as antiviral, antibacterial antifungal, antiparasitic, antitumour, transferrin transporters, and anti-inflammatory agents are being intensively investigated. In this study we examined the antifungal activity of Co(III) complexes with diamine chelate ligands against a broad spectrum of *Candida* species. Minimum inhibitory concentration was determined by the microbroth dilution method and with serial passaging assay; the synergistic antimicrobial activity of the tested complexes combined with two antifungal drugs (ketoconazole and amphotericin B) was made by checkerboard assay. The effects of Co(III) complexes on yeast cell morphology were studied by optical and transmission electron microscopy. The mode of action of Co(III) complexes on the yeast cell wall (sorbitol assay) and cell membrane (ergosterol assay) were investigated. The cytotoxic effects of the tested compounds on red blood cells and the human keratinocyte (HaCaT) cell line were also evaluated. The analyzed compounds revealed significant antifungal activity for selected strains of *Candida* species; [CoCl_2_(dap)_2_]Cl (1) and [CoCl_2_(en)_2_]Cl (2) were more effective than ketoconazole. Its probable mechanism of action did not involve the cell wall or ergosterol binding. However, the checkerboard assay showed, that the antifungal activity of ketoconazole increased in combination with the tested complexes of Co(III). Our results suggest that both diamine complexes with Co(III) analogs caused damage to mitochondrial membrane or the membrane of the endoplasmic reticulum. The effect was observed by transmission electron microscope. Co(III) complexes with diamine chelate ligands are non-toxic at concentrations active against *Candida* species. This study provides new data on potential antifungal drugs, especially against *Candida* species.

## Introduction

*Candida* spp., in particular *Candida albicans*, is one of the most important opportunistic fungal pathogens, which can harmlessly colonize the gastrointestinal tract, mouth, skin and urogenital system (Wu et al., 2003; Jackson et al., 2007; Achkar and Fries, 2010; Rosenbach et al., 2010; Naglik et al., 2011). However, it can also cause infections, especially among people with weakened immune systems, attacking the skin, mucous membranes, getting into the blood, and attacking internal organs. Risk factors that are conducive to the development of systemic infections caused by *Candida* include: long-term stay in intensive care units, surgery (especially operations in the abdominal cavity), broad-spectrum antibiotic intake, and immunosuppressants (Kontoyiannis et al., 2003; Pfaller and Diekema, 2004; Zaoutis et al., 2005; Sydnor and Perl, 2011). Antitumor chemotherapy, organ transplants, haemodialysis, parenteral nutrition, or venous catheters contribute to the invasion of fungi (Mikulska et al., 2012). Other species of *Candida* increasingly isolated from patients are *Candida glabrata*, *Candida tropicalis*, *Candida parapsilosis*, and *Candida krusei* (MacCallum, 2012).

In the case of infections, *Candida* mortality can reach 45%. This is probably due to the inappropriate use of diagnostic methods, improper treatment or improper doses of drugs (Morrell et al., 2005). Despite the availability of treatment using antifungal agents, the high morbidity and mortality rates associated with candidiasis (Pfaller and Diekema, 2007) and the growing resistance of yeast to antifungals requires the development of novel compounds with novel mechanisms of action. This would facilitate treatment that could combat and effectively control these microorganisms. The search for a drug acting on specific targets present in the fungal cell is very difficult because fungi such as *Candida* spp. are eukaryotic and thus share many features common with mammalian cells (Ostrosky-Zeichner et al., 2010; Roemer and Krysan, 2014). Consequently, the list of antifungal agents used currently in clinical therapy for the treatment of infections caused by *Candida* is limited (e.g., polyenes, azoles or echinocandins, presently considered to be the most effective in antifungal therapy) (Andriole, 1999; Pierce et al., 2013; Roemer and Krysan, 2014). Major targets for potential antifungal agents are: (1) ergosterol (inhibiting its biosynthesis or binding to it), an essential lipid of the yeast cell membrane (not present in mammalian cells); and (2) chitin and β-glucan (inhibition of its synthesis), key structural components of the fungal cell wall (Hof, 2006; Sundriyal et al., 2006; Pierce et al., 2013; Ngo et al., 2016).

The intensive use of available drugs has led to the emergence of resistant strains of fungi. The most common resistance mechanisms are: overexpression of efflux pump proteins, mutations on the target enzyme (e.g., lanosterol demethylase) or biofilm formation (Perea et al., 2001; Ramage et al., 2001; Kuhn and Ghannoum, 2004). The side effects of drugs in therapeutic doses (e.g., amphotericin B) in turn has led to serious health problems after their use (Groll et al., 1998; Mahmound and Louis, 1999; Sanglard and Odds, 2002; Pierce et al., 2013). Due to the short list of antifungal agents, efforts have been made to improve the effectiveness or to reduce the toxicity of drugs, e.g., through obtaining a synergistic effect by the combined application of antifungals (e.g., the combination of fluconazole and amphotericin B) (Odds, 2003a). Moreover, modifications to the chemical structures of molecules of available antifungals have improved their activity and pharmacokinetic parameters (Scorzoni et al., 2017). Simultaneously, completely new chemical compounds with an alternative mode of action, high antifungal activity and low toxicity are being sought. In the field of antifungals, amino acid biosynthesis inhibitors appear to be a very promising group of compounds (Jastrzębowska and Gabriel, 2015).

**FIGURE 1:**
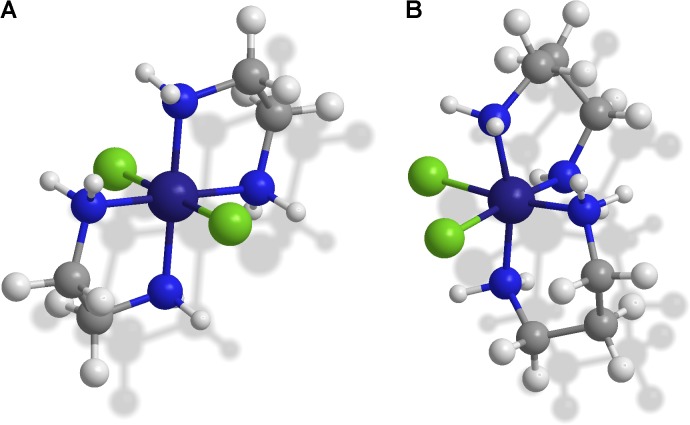
The spatial presentation of the selected trivalent Co complex cation structures as optical isomers: **(A)** *trans*-[CoCl_2_(en)_2_]^+^ with green color as a solid state turning to purple after spontaneous isomerization to *cis* configuration occurring in an aqueous solution; **(B)** *cis*-[CoCl_2_(dap)_2_]^+^ with purple color as an aqueous solution turning to dark blue after isomerization to *trans* configuration stabilized in solid state.

A very interesting and attractive group of compounds seems to be complexes of Co(III) with diamine chelate ligands. The main feature allows the use of complexes of cobalt(III) as a component of chemotherapeutic agents is the presence of stable Co(III) and labile Co(II). On the other hand, complexes of Co(II) are stable in solid form but exhibit remarkable ease of oxidation under biological conditions. The antiviral, antibacterial, antitumour and antifungal activities of Co(II) and Co(III) coordination compounds has been widely described (Arali et al., 1993; Yadave and Patil, 1997; Walker et al., 2003; Yilmaz and Cukurovali, 2003; Liang et al., 2004; Belicchi-Ferrari et al., 2005; Lv et al., 2006; Zhong et al., 2006; Bisceglie et al., 2007; Penumaka and Satyanarayana, 2007; Konidaris et al., 2010). Interestingly, the cobalt(III) complexes are also being investigated regarding their anticancer activity, including the identification of cytostatic factors characterized by strong interactions with cancer cells and very weak effects on the body’s healthy cells (Brown, 1999; Dachs and Tozer, 2000). Two types of coordination complexes [CoCl_2_(N,N)_2_]Cl, where N,N is ethylenediamine or 1,3-diaminopropane (**Figure 1**) as the chelating moiety, are new variants of this class of chemical compound, and current knowledge about their biological activity is limited to preliminary results. Recent studies involving biological tests with coordination compounds of Co(III) with N,N-donor organic ligands have revealed antibacterial and antifungal activity (Chylewska et al., 2013).

In the present work we determined the antifungal activity of Co(III) complexes with diamine chelate ligands against a wide range of *Candida* spp. alone and in combination with standard antifungal drugs. In addition, we examined the effect of compounds on fungal morphology and the mechanism of antifungal action using light and electron microscopy. We also conducted tests to assess the toxicity of Co(III) compounds.

## Materials and Methods

### Strains, Media, and Growth Conditions

The reference and clinical strains used in the study are listed in **Table 1**. The isolates originated from the collection of the Department of Pharmaceutical Technology and Biochemistry, Faculty of Chemistry, Gdańsk University of Technology. Strains were stored as glycerol stock at -70°C. For research purposes cultures were conducted at 28°C for 24 h in RPMI 1640 broth (Sigma-Aldrich, Poland).

**Table 1:** Strains used in the study.

Organism	Strain designation	Origin
*Candida albicans*	ATCC 10231	Reference
*Candida albicans*	12823	Clinical
*Candida albicans*	Flu^R^96	Clinical, fluconazole resistant
*Candida albicans*	Flu^R^79	Clinical, fluconazole resistant
*Candida albicans*	Flu^S^81	Clinical, fluconazole sensitive
*Candida albicans*	12588	Clinical
*Candida albicans*	12900	Clinical
*Candida albicans*	1600/1	Clinical
*Candida glabrata*	ATCC 2001	Reference
*Candida glabrata*	11644	Clinical
*Candida glabrata*	DSM 11226	Clinical
*Candida krusei*	ATCC 6258	Reference
*Candida krusei*	Flu^S^59	Clinical, fluconazole sensitive
*Candida krusei*	Flu^R^176	Clinical, fluconazole resistant
*Candida tropicalis*	12946	Clinical
*Candida parapsilosis*	ATCC 22019	Reference
*C. lusitaniae*	ATCC 34449	Reference


### Synthesis of *trans*-[CoCl_2_(N,N)_2_]Cl

The synthesis of *trans-*[CoCl_2_(dap)_2_]Cl **(1)** and *trans-*[CoCl_2_(en)_2_]Cl **(2)** was performed according to the procedure described by Chylewska et al. (30).

Compound **(1)**: *trans*-[CoCl_2_(dap)_2_]Cl: molar mass: 349.57 g/mol; blue crystals, very soluble in water (pink solution, isomerizes from blue *trans*- to pink *cis*-). Compound **(2)**: *trans-*[CoCl_2_(en)_2_]Cl molar mass: 226.5 g/mol; dark green crystals, very soluble in water (a green solution isomerizes from blue *trans*- to pink *cis*-).

### Determination of the Minimum Inhibitory Concentration (MIC) and Minimum Fungicidal Concentration (MFC)

The MIC and MFC of commercial drugs (ketoconazole and amphotericin B) and tested coordination compounds of Co(III) were determined by the microbroth dilution method using 96-well plates according to Clinical and Laboratory Standards Institute (Method M27-A3, Clinical and Laboratory Standards Institute [CLSI], 2008) against reference and clinical strains of *Candida.* Ketoconazole and amphotericin B (Sigma-Aldrich) were dissolved in DMSO (dimethyl sulfoxide) to give a stock concentration of 6400 µg/mL. Crystals of Co(III) compounds were dissolved in sterile water to give a stock concentration of 32,000 µg/mL. The culture of yeasts were prepared by transferring cells from the stock cultures to tubes with RPMI 1640 broth and incubated with agitation for 24 h at 28°C. The cultures were diluted with the same broth to achieve an optical density corresponding to 10^3^ colony-forming units per mL (CFU/mL). After filling each well with 100 µL of broth, water solutions of tested coordination compounds were added to the first well of each microtiter line. Dilution in geometric progression was done by transferring the dilution (100 µL) from the first to twelfth well. An aliquot in volume of 100 µL was discarded from the twelfth well. In case of DMSO solutions 96 µL of RPMI 1640 broth and 4 µL of appropriate concentration of antifungal drugs were added to each well. The fungal suspension (100 µL) at 10^3^ CFU/mL was added to each well. The final concentration of the tested compounds ranged from 128 to 0.0625 µg/mL (DMSO solutions) and from 8000 to 3.9 µg/mL (water solutions). The growth controls of yeasts in the medium without antifungal compounds were also carried out. Plates were incubated at 28°C for 24 h and then fungal growth was assessed. The MIC was determined as the lowest concentration of the sample that demonstrated no visible growth [the lowest drug concentrations that caused complete growth inhibition (100%) compared with control probes without antifungal compounds] (Espinel-Ingroff et al., 2016). Next, 100 µL of the suspension from each well without growth was inoculated on agar plates as a control of yeast viability. After incubation 48 h at 28°C, the CFU was determined. The MFC (minimal fungicidal concentration) was defined as the minimal concentration of tested compounds required to kill 99.9% the organisms (Hałasa et al., 2014).

### Investigation of Synergy of Commercial Antibiotics and Complexes of Co(III)

To determine the fractional inhibitory concentrations (FICs) of standard antifungal drugs (amphotericin B and ketoconazole, Sigma–Aldrich) in combination with Co(III) complexes a checkerboard assay (CLSI) was used in 96 well microtiter plates. The concentration ranges for antifungal drugs were from 128 to 0.125 µg/mL and for Co(III) complexes from 500 to 7.8 µg/mL. Each well in a 96-well plate was inoculated with 100 µg/mL of fungal inoculum of 1 × 10^3^ CFU/mL. Next plates were incubated at 28°C for 24–48 h. Each test was performed in triplicate. After describing the MIC for each row the FIC was calculated as: ΣFIC = FIC_A_ + FIC_B_ = (C_A_/MIC_A_) + (C_B_/MIC_B_), where MIC_A_ and MIC_B_ are the MIC_S_ of drugs A and B alone, respectively, and C_A_ and C_B_ are the concentrations of the drugs in combinations, respectively. FIC(index) values were interpreted as follows (Meletiandis et al., 2005): FIC ≤ 0.5, synergistic; FIC > 0.5 and <4, no interaction; FIC > 4, antagonistic (Odds, 2003b).

### Transmission Electron Microscopy

Overnight cultures of *Candida* spp. grown on RPMI 1640 broth were diluted with the same broth to 0.5 McFarland. The tested compounds were added to 5 mL of the yeast suspension in the appropriate concentration (equivalent to MIC) and then incubated at 28°C for 24 h. Following the incubation period probes were centrifuged and fixed overnight in 2.5% glutaraldehyde (Polysciences, Warrington, PA, United States) in PBS (phosphate-buffered saline), washed three times in PBS and postfixed overnight in 1% osmium tetroxide (Polysciences, Warrington, PA, United States). Following ethanol dehydration, probes were embedded in Epon resin (Sigma-Aldrich), cut on a ultramicrotome Leica UC7 and contrasted in uranyl acetate and lead citrate. Transmission electron microscopy studies were performed using a Philips CM100 electron microscope (Banasiuk et al., 2016).

### Trypan Blue Assay

Overnight cultures of *Candida* spp. grown on RPMI 1640 broth were diluted with the same broth to 0.5 McFarland. The tested compounds were added to 1 mL of the yeast suspension in appropriate concentrations (equivalent to MIC) and were incubated at 28°C for 24 h. After the incubation time, the suspension of yeast cells were diluted in 0.4% trypan blue dye solution in a dilution ratio 1:1. Samples were left for 2 min, then cell viability was evaluated using optical microscope Nikon Eclipse E600. Non-viable cells were blue, viable cells were unstained.

### Sorbitol Assay

To test the effect of Co(III) coordination compounds on the cell wall of *Candida* spp., the sorbitol assay was performed. The MICs of investigated compounds were determined in the presence of sorbitol according to CLSI method (Method M27-A3, Clinical and Laboratory Standards Institute [CLSI], 2008). After filling each well of the microplates with 100 µL of Sabouraud Dextrose Broth (SDB), serial dilutions of Co(III) complexes ranging from 8000 to 3.9 µg/mL were carried out. In the case of DMSO solutions, 96 µL of Sabouraud Dextrose broth and 4 µL of the appropriate concentration of antifungal drugs (amphotericin B and ketoconazole) were added to each well. Next, 100 µL of inoculum (10^3^ CFU/mL) prepared in SDB supplemented with sorbitol at a final concentration of 0.8 M (Sigma-Aldrich) was introduced into each well (Pereira et al., 2016). Probes were incubated at 28°C for 2 and 7 days.

### Ergosterol Assay

Ergosterol was prepared according to the procedure described by Leite et al. (2014) at the time of test execution and dissolved in DMSO and Tween 80 in accordance with the desired concentration and volume. The formed emulsion was homogenized, heated and diluted with the RPMI 1640 medium. The MIC of Co(III) complexes and amphotericin B (as a positive control drug which binds to ergosterol in the fungal membrane) against *Candida* strains was determined by the microdilution method, in the presence and absence of exogenous ergosterol (Sigma-Aldrich). The concentrations of amphotericin B ranged from 128 to 0.125 µg/mL and of Co(III) complexes from 7.8 to 500 µg/mL in RPMI 1640 medium (100 µL) supplemented with ergosterol at a concentration of 400 µg/mL. Yeast suspension (10 µL; 05 McFarland) was added to each well and plates were incubated at 35°C for 24 h. The MIC was determined as the lowest sample concentration that prevented visible growth.

### Erythrocyte Lysis Assay

Erythrocytes were harvested from 5.0 mL fresh sheep blood (BioMaxima S.A., Center for Microbiology, Emapol, Gdańsk, Poland) by centrifugation for 10 min at 1000 × *g* and washed three times with 0.9% NaCl. Cell suspension (with final concentration of 10^8^ cells/mL) in a volume of 100 µL was added to each well of a 96-well microtiter plate and incubated at 37°C in the presence of tested compounds in the concentration range from 4000 to 0.008 µg/mL (diluted in saline solution). For comparison purposes amphotericin B and ketoconazole in the concentration range of 128–0.125 µg/mL were also examined. To estimate the relative haemolytic potential of the tested compounds, the appropriate controls, i.e., 100% erythrocyte lysis using 4% Triton X-100 and 0% lysis in saline solution were used. Plates with samples were incubated for 1 h at 37°C, then centrifuged for 10 min at 1000 × *g* to separate the unlysed erythrocytes and subsequently the supernatant was transferred to a new plate. The absorbance was measured spectrophotometrically at 450 nm. The hemolysis percentage was calculated according to the equation presented by Sharma et al. (2016):

% hemolysis = [(A450 of test compound treated sample-A450 of buffer treated sample)/(A450 of 4% Triton X-100 treated samples-A450 of buffer treated sample)] × 100 (Sharma et al., 2016).

### Cytotoxicity Assay

The cytotoxicity studies were performed on the human keratinocyte (HaCaT) cell line (obtained from the Department of Medical Biotechnology of the Intercollegiate Faculty of Biotechnology, University of Gdańsk, Medical University of Gdańsk, Poland). The HaCaT cell line was cultured in Dulbecco’s modified Eagle’s medium supplemented with 2 mM glutamine, 10% fetal bovine serum, 100 µg/mL streptomycin and 100 units/mL penicillin (Sigma-Aldrich). The cells were cultured in a humidified atmosphere with 5% CO_2_ at 37°C.

The cytotoxicity assay was performed with the HaCaT cell line using the MTT [3,(4,5-dimethylthiazol-2-yl)-2,5-diphenyltetrazolium bromide] test (Mossman, 1983). This is a reference test to determine the metabolic activity of cells based on their mitochondrial oxidative activity. The MTT assay is based on the ability of mitochondrial dehydrogenase to convert yellow water-soluble tetrazole salt to a purple insoluble formazan in living cells. The cells in the logarithmic growth phase were seeded in 96-well microtiter plates (5 × 10^4^ cells per well) and treated for 24 h with tested compounds in the concentration range of 0–4000 µg/mL. MTT was added to each well and incubated for 3 h at 37°C following lysis of cells with dimethylsulfoxide. Optical density of the purple formazan solution was measured by spectrophotometry at 550 nm with plate reader (Victor, 1420 multilabel counter). The amount of colored reduced MTT is proportional to the number of metabolically active cells (living cells in the population) (Sykłowska-Baranek et al., 2012).

### Serial Passages Assay

Four strains of *Candida* spp. (*C. albicans* ATCC 10231, *C. albicans* 12823, *C. glabrata* ATCC 2001 and *C. glabrata* 11644) were selected for the serial passages studies. The suspension of yeasts (10 µL) in RPMI 1640 medium (approximately 10^8^ CFU/mL) was incubated in the presence of compounds ranging from the four doubling dilutions above (4MIC, 2MIC) to three doubling dilutions below the MIC (1/2MIC, 1/4MIC, and 1/8MIC) at 28°C for 24 h. Next, 10 µL of the appropriate inoculums of yeast with a compound was transferred to a medium containing the same amount of the compound. The above-described steps were repeated 20 times. After each passage MIC values for chosen strains were determined (Kumar and Shukla, 2010).

### Stability Assay

The MIC and MFC values of the tested coordination complexes of Co(III) were determined within 14 and 30 days after preparation of fresh solutions of compounds against reference strains *C. albicans* ATCC 10231 and *C. glabrata* ATCC 2001. During the test the compounds were maintained at a temperature of 25–28°C under dark and light conditions.

### Statistical Analysis

All experiments were performed in triplicates, in three independent experimental sets. The graphics and the data were constructed and analyzed statistically by means ± SD.

## Results

### Minimal Inhibitory Concentration (MIC) and Minimal Fungicidal Concentration (MFC) Assays

The antimicrobial activity of the coordination compounds of Co(III) with N,N’-donor organic ligands was tested using the microbroth dilution method. For comparison purposes amphotericin B and ketoconazole were also examined. A broad spectrum of reference and clinical strains of *Candida* was analyzed. The tested compounds inhibited the growth of all studied strains and there was no difference in sensitivity between reference and clinical strains of yeast within the species. The MIC_S_ of **(1)** and **(2)** were within the range of 16–125 µg/mL (4.6–715 µM/L). The most sensitive proved to be *C. glabrata* [MIC 16 µg/mL (4.6 µM/L)] (**Table 2**). The fungicidal effect ranged from 31.25 to 500 µg/mL (89–1430 µM/L) for **(1)** and 31.25–1000 µg/mL (89–4420 µM/L) for **(2)**. The most resistant proved to be the strain of *C. parapsilosis* [MIC 125 (715 µM/L) and 250 µg/mL (1103 µM/L) for compound 1 and 2, respectively].

**Table 2:** MIC and MFC values in µg/mL (µM/L) of [CoCl_2_(dap)_2_]Cl (1), [CoCl_2_(en)_2_]Cl (2), amphotericin B and ketoconazole against *Candida* strains.

Strains	[CoCl_2_(dap)_2_]Cl (1)		[CoCl_2_(en)_2_]C (2)		Amphotericin B		Ketoconazole	
				
	MIC	MFC	MIC	MFC	MIC	MFC	MIC	MFC
*C. albicans* ATCC 10231	62.5 ± 4.95	125 ± 9.89	62.5 ± 4.039	125 ± 8.08	0.5 ± 0.06	0.5 ± 0.08	>128	>128
	(179)	(715)	(276)	(552)	(0.54)	(0.54)	>(241)	>(241)
*C. albicans* 12823	62.5 ± 4.04	250 ± 16.16	62.5 ± 4.95	250 ± 19.79	0.5 ± 0.06	>128	128 ± 16.54	>128
	(179)	(720)	(276)	(1104)	(0.54)	>(241)	(241)	>(241)
*C. albicans* Flu^R^96	62.5 ± 4.95	250 ± 16.16	125 ± 8.08	500 ± 32.31	1 ± 0.06	2 ± 0.13	128 ± 16.54	>128
	(179)	(720)	(552)	(2210)	(1.1)	(2.2)	(241)	>(241)
*C. albicans* Flu^S^79	62.5 ± 4.04	125 ± 16.16	125 ± 9.89	500 ± 32.31	2 ± 0.16	4 ± 0.52	>128	>128
	(179)	(715)	(552)	(2210)	(2.2)	(4.3)	(241)	>(241)
*C. albicans* Flu^S^81	62.5 ± 8.08	125 ± 9.89	125 ± 8.08	250 ± 16.16	1 ± 0.13	2 ± 0.27	128 ± 16.54	>128
	(179)	(715)	(552)	(11040)	(1.1)	(2.2)	(241)	>(241)
*C. albicans* 12588	16 ± 1.97	250 ± 32.31	62.5 ± 8.08	1000 ± 64.63	0.5 ± 0.08	0.5 ± 0.07	>128	>128
	(4.6)	(720)	(276)	(4420)	(0.54)	(0.54)	>(241)	>(241)
*C. albicans* 12900	125 ± 8.08	500 ± 32.31	125 ± 9.89	1000 ± 79.15	2 ± 0.16	4 ± 0.26	128 ± 20.26	>128
	(715)	(1430)	(552)	(4420)	(2.2)	(4.3)	(241)	>(241)
*C. albicans* 1600/1	62.5 ± 8.08	250 ± 32.31	125 ± 9.89	250 ± 39.58	0.125 ± 0.02	0.5 ± 0.04	>128	>128
	(179)	(720)	(552)	(1104)	(0.14)	(0.27)	>(241)	>(241)
*C. glabrata* ATCC 2001	16 ± 1.97	62.5 ± 8.08	16 ± 2.41	62.5 ± 8.08	0.25 ± 0.02	0.5 ± 0.06	64 ± 5.07	128 ± 16.54
	(4.6)	(179)	(70.6)	(276)	(0.27)	(0.54)	(120)	(241)
*C. glabrata* 11644	16 ± 2.41	31.25 ± 4.04	16 ± 1.97	31.25 ± 4.04	0.5 ± 0.04	2 ± 0.13	64 ± 4.14	>128
	(4.6)	(89)	(70.6)	(380)	(0.54)	(2.2)	(120)	>(241)
*C. glabrata* DSM 11226	31.25 ± 4.04	125 ± 18.66	62.5 ± 6.35	250 ± 32.31	0.5 ± 0.04	1 ± 0.13	128 ± 10.13	>128
	(89)	(715)	(276)	(1104)	(0.54)	(1.1)	(241)	>(241)
*C. krusei* ATCC6258	62.5 ± 4.95	125 ± 16.16	62.5 ± 4.04	250 ± 32.31	2 ± 0.16	4 ± 0.63	128 ± 8.27	>128
	(179)	(715)	(276)	(104)	(2.2)	(4.3)	(241)	>(241)
*C. krusei* Flu^S^59	62.5 ± 4.04	125 ± 16.16	62.5 ± 4.95	125 ± 8.08	2 ± 0.16	8 ± 0.63	128 ± 10.13	>128
	(179)	(715)	(276)	(552)	(2.2)	(8.7)	(241)	>(241)
*C. krusei* Flu^R^176	62.5 ± 4.95	62.5 ± 4.95	62.5 ± 4.95	62.5 ± 4.04	1 ± 0.13	4 ± 0.26	128 ± 8.27	>128
	(179)	(179)	(276)	(276)	(1.1)	(4.3)	(241)	>(241)
*C. tropicalis* 12946	31.25 ± 4.04	125 ± 9.89	31.25 ± 4.04	250 ± 16.16	1 ± 0.16	4 ± 0.26	128 ± 8.27	>128
	(89)	(715)	(138)	(1104)	(1.1)	(4.3)	(241)	>(241)
*C. parapsilosis* ATCC 22019	125 ± 9.89	500 ± 39.58	250 ± 16.16	1000 ± 79.15	2 ± 0.13	4 ± 0.52	32 ± 5.07	64 ± 8.27
	(715)	(1430)	(1103)	(4420)	(2.2)	(4.3)	(60)	(241)
*C. lusitaniae*	62.5 ± 8.08	500 ± 39.57	125 ± 16.16	500 ± 32.31	2 ± 0.16	4 ± 0.52	128 ± 10.13	>128
	(179)	(1430)	(552)	(2210)	(2.2)	(4.3)	(241)	>(241)


*Data shown are mean ± SD.*

Although the tested complexes were active, they did not reach the level of effectiveness of the conventional antifungal drug – amphotericin B. However, in case of most of *C. albicans* strains, *C. glabrata*, and *C. krusei* have been found to be more effective than ketoconazole (**Table 2**).

### Light Microscope Assay

**FIGURE 2:**
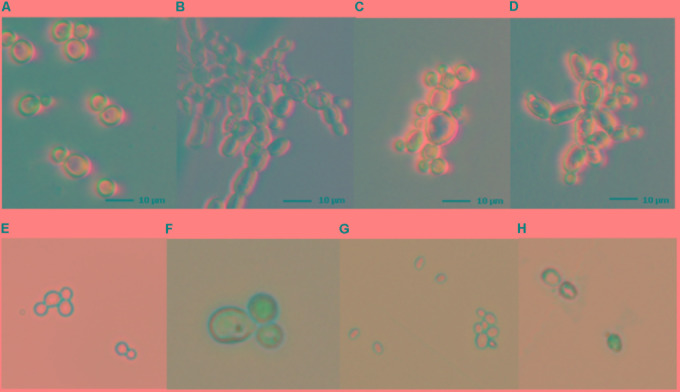
Effect of Co(III) complexes with diamine chelate ligands and amphotericin B on *Candida* spp. cells **(A–D)** and evaluation of *Candida* cell viability **(E–H)**. Light microscopy images of the *C. albicans* strains: *C. albicans* ATCC 10231 in the absence **(A)** and presence of amphotericin B **(B)**, [CoCl_2_(dap)_2_]Cl **(C)** and [CoCl_2_(en)_2_]Cl **(D)** complexes in MIC values; *C. albicans* ATCC 10231 **(E,F)** and *C. glabrata* ATCC 2001 **(G,H)** strains stained with trypan blue in the absence **(E,G)** and presence of [CoCl_2_(dap)_2_]Cl complex **(F,H)** in MIC values. Magnification 400×.

The analysis of the effect of Co(III) complexes with diamine chelate ligands on the morphology of *C. albicans* (ATCC and 12823) strains were based on optical microscope observations. These studies showed morphological changes in yeast cells after treatment with **(1)** and **(2)** complexes in MIC concentrations. In contrast with untreated cultures of *C. albicans* (**Figure 2A**), visible changes in the shape of cells treated with the mentioned complexes were observed (**Figures 2C,D**). In addition, a tendency for cell aggregation has been recorded. The same effect was observed in a control study with amphotericin B (**Figure 2B**).

### Trypan Blue Assay

The viability of the yeast cells was determined by staining reference and clinical *C. albicans* and *C. glabrata* strains with trypan blue. Cells treated with Co(III) complexes stained blue, indicating changes in membrane permeability and cell death. In contrast, control cells, not treated with tested complexes, did not stain with trypan blue, indicating live cells with intact cell membranes (**Figures 2E–H**).

### Transmission Electron Microscopy Assay

**FIGURE 3:**
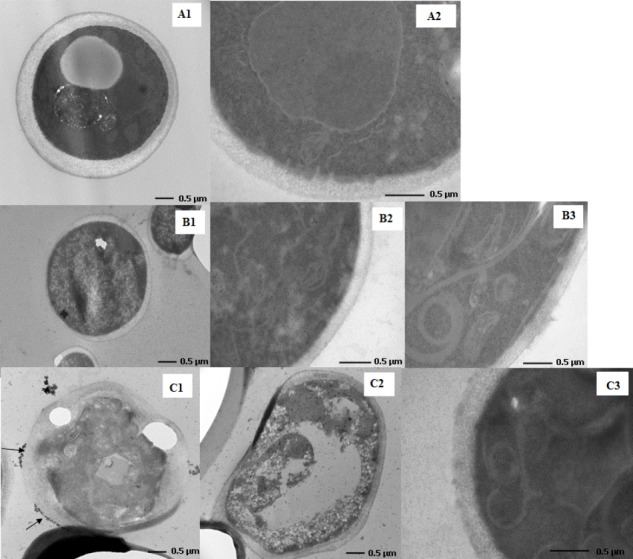
Changes in the cell morphology of compound 1- and compound 2-treated *Candida albicans.* Transmission electron microscopy images of *C. albicans* ATCC 10231 strain in the absence **(A1,A2)** and presence [CoCl_2_(dap)_2_]Cl **(B1–B3)** and [CoCl_2_(en)_2_]Cl **(C1–C3)** complexes in MIC values.

Studies using Transmission Electron Microscopy (TEM) exhibited well visible contraction of the fungal cells after the application of the tested complexes both in the case of reference and clinical (results not shown) strains of yeasts. The regular internal structures of *C. albicans* strains can be observed in the control cells. The regular cellular wall, plasma membrane, endoplasmic reticulum, mitochondria, nucleus and vacuole were clearly visible. In contrast with untreated growth control, disorganization of internal structures were observed in the fungal cell treated with the tested compounds (**Figure 3**). After exposure to the MIC of compound 1 and compound 2 we observed yeast cell deformation and the disruption of internal organelle membranes. Some cellular organelles were damaged, such as mitochondria or endoplasmic reticulum (**Figures 3B2,B3,C3**). Plasma membrane became irregular (**Figures 3C1,C2**) and the cell wall varied in thickness at certain sites (**Figure 3B1**). It was also visible that cells were coated probably with tested compounds (black arrows). Changes in yeast cells morphology can suggest disorders in cell membrane permeability.

### Investigation of Synergy of Commercial Antibiotics and Complexes of Co (III)

The combination of both diamine complexes with Co(III) analogs and amphotericin B or ketoconazole was also assayed for their effect on the growth of *Candida* spp. by the checkerboard titration method.

We observed a significant reduction of MICs of ketoconazole against *C. albicans* strains. In combination with **(1),** ketoconazole was able to inhibit *C. albicans* ATCC 10231 strain growth at 2 µg/mL and *C. albicans* clinical strain (12823) at 1 µg/mL, which was 128 times lower than MIC of ketoconazole alone. While, in the case of the combination of ketoconazole with **(1)** against *C. krusei* strains, inhibition of growth was observed at 1 µg/mL (*C. krusei* ATCC 6258), 4 µg/mL (*C. krusei* Flu^S^59), and 32 µg/mL (*C. krusei* Flu^R^176), which means lowering the MIC value 128, 32, and 4 times, respectively. Also, the same combination of antifungals against *C. tropicalis* showed a reduction of the MIC value 64 times (MIC at 2 µg/mL). In combination with **(2)** ketoconazole was able to inhibit *C. albicans* ATCC 10231 strain growth at 0.125 µg/mL and *C. albicans* clinical strain (12823) at 0.25 µg/mL, which indicates a 2048 and 256 times lower MIC, respectively, than that of ketoconazole alone. To quantitatively determine the interaction between the tested complexes of Co(III) and the chosen antifungal drugs, FIC values were calculated according to the formula given in Material and Methods (**Tables 3**, **4**). Compound **(1)** showed synergistic activity with ketoconazole against *C. albicans* ATCC 10231 (FIC 0.51), *C. albicans* 12823 (FIC 0.29), *C. krusei* ATCC 6258 (FIC 0.38) *C. krusei* Flu^S^59 (FIC 0.29), *C. krusei* Flu^R^176 (FIC 0.50), and *C. tropicalis* (FIC 0.51). In the case of **(2)** the same effect against *C. albicans* ATCC 10231 and *C. albicans* 12823 was observed for both strains, FIC 0.26. The combination of tested compounds with amphotericin B was found to be non-interfering.

**Table 3:** Fractional inhibitory concentration index (FICi) values of [CoCl_2_(dap)_2_]Cl (1), amphotericin B and ketoconazole.

Strains	Origin	Antifungal agent	MIC of Antifungal agent (µg/mL)		MIC of (1) (µg/mL)		FICi	Outcome
						
			Alone	Com.	Alone	Comb.		
*Candida albicans*	Reference	Amphotericin B	0.5	0.125	62.5	31.25	0.63	No interaction
ATCC 10231		Ketoconazole	256	2	62.5	31.25	0.51	Synergy
*Candida albicans*	Clinical	Amphotericin B	0.5	0.125	62.5	16	0.76	No interaction
12823		Ketoconazole	128	1	62.5	16	0.26	Synergy
*Candida glabrata*	Reference	Amphotericin B	0.25	0.25	16	31.25	2.0	No interaction
ATCC 2001		Ketoconazole	64	>128	16	31.25	2.0	No interaction
*Candida glabrata*	Clinical	Amphotericin B	0.5	0.25	16	7.8	1.49	No interaction
11644		Ketoconazole	64	16	16	7.8	0.74	No interaction
*Candia krusei*	Reference	Amphotericin B	2	0.25	62.5	31.25	0.75	No interaction
ATCC 6258		Ketoconazole	128	1	62.5	16	0.26	Synergy
*Candida krusei*	Clinical	Amphotericin B	2	0.5	62.5	31.25	1.05	No interaction
Flu^S^59		Ketoconazole	128	4	62.5	16	0.29	Synergy
*Candida krusei*	Reference	Amphotericin B	1	0.125	62.5	16	0.76	No interaction
Flu^R^176		Ketoconazole	128	32	62.5	16	0.50	Synergy
*Candida tropicalis*	Clinical	Amphotericin B	1	0.5	31.25	16	1.5	No interaction
		Ketoconazole	128	2	31.25	16	0.51	Synergy


**Table 4:** Fractional inhibitory concentration index (FICi) values of [CoCl_2_(en)_2_]Cl (2), amphotericin B and ketoconazole.

Strains	Origin	Antifungal agent	MIC of Antifungal agent (µg/mL)		MIC of (2) (µg/mL)		FICi	Outcome
						
			Alone	Com.	Alone	Comb.		
*Candida albicans*	Reference	Amphotericin B	0.5	0.125	62.5	32	0.76	No interaction
ATCC 10231		Ketoconazole	256	0.125	62.5	16	0.26	Synergy
*Candida albicans*	Clinical	Amphotericin B	0.25	0.125	62.5	16	0.76	No interaction
12823		Ketoconazole	64	0.25	62.5	16	0.26	Synergy
*Candida glabrata*	Reference	Amphotericin B	0.25	0.25	16	16	2.0	No interaction
ATCC 2001		Ketoconazole	64	8	16	16	1.13	No interaction
*Candida glabrata*	Clinical	Amphotericin B	0.25	0.25	16	7.8	1.49	No interaction
11644		Ketoconazole	64	8	16	7.8	0.61	No interaction
*Candia krusei*	Reference	Amphotericin B	0.5	0.25	62.5	31.25	1.00	No interaction
ATCC 6258		Ketoconazole	128	4	62.5	31.25	0.53	No interaction
*Candida krusei*	Clinical	Amphotericin B	1	0.5	62.5	31.25	1.00	No interaction
Flu^S^59		Ketoconazole	128	4	62.5	31.25	0.53	No interaction
*Candida krusei*	Reference	Amphotericin B	1	0.5	62.5	31.25	1.00	No interaction
Flu^R^176		Ketoconazole	128	16	62.5	31.25	0.63	No interaction
*Candida tropicalis*	Clinical	Amphotericin B	1	0.5	31.25	16	1.01	No interaction
		Ketoconazole	128	2	31.25	16	0.53	No interaction


### Sorbitol Assay

Sorbitol acts as a substance that maintains proper osmotic pressure, thereby providing a suitable environment for the cell wall biosynthesis pathway. The MIC of the coordination compounds of Co(III) with N,N-donor organic ligands in the presence and absence of 0.8 M sorbitol was analyzed. The viability of control strains containing only yeast and sorbitol in a culture medium was also examined. It was found that the there are no significant differences between MIC of the tested compounds against reference and clinical strains of *Candida* in either the absence or presence of sorbitol (**Table 5**), while the control sample showed the ability to grow in the presence of sorbitol.

**Table 5:** MIC (µg/mL) values in the presence of sorbitol and ergosterol against different strains of *Candida* spp.

Strain	Compound	Sorbitol			Ergosterol	
			
		Absence	Presence		Absence	Presence
					
			2 d^∗^	7 d^∗^		
*C. albicans* ATCC 10231	[CoCl_2_(dap)_2_]Cl (1)	62.5 ± 4.95	62.5 ± 4.95	125 ± 8.08	62.5 ± 4.04	62.5 ± 4.95
	[CoCl_2_(en)_2_]Cl (2)	62.5 ± 8.08	125 ± 9.98	125 ± 9.89	62.5 ± 4.04	62.5 ± 8.08
	Amphotericin B	–	–	–	0.5 ± 0.04	64 ± 4.13
*C. albicans* 12823	[CoCl_2_(dap)_2_]Cl (1)	62.5 ± 4.04	125 ± 9.98	250 ± 16.16	62.5 ± 4.04	125 ± 9.89
	[CoCl_2_(en)_2_]Cl (2)	62.5 ± 4.95	250 ± 19.79	250 ± 19.79	62.5 ± 4.95	125 ± 8.08
	Amphotericin B	–	–	–	0.5 ± 006	64 ± 5.07
*C. glabrata* ATCC 2001	[CoCl_2_(dap)_2_]Cl (1)	16 ± 1.97	31.25 ± 1.97	62.5 ± 8.47	16 ± 2.41	16 ± 1.97
	[CoCl_2_(en)_2_]Cl (2)	16 ± 1.29	31.25 ± 4.04	62.5 ± 4.95	16 ± 2.41	16 ± 2.41
	Amphotericin B	–	–	–	0.25 ± 0.02	32 ± 2.53
*C. glabrata* 11644	[CoCl_2_(dap)_2_]Cl (1)	16 ± 1.06	31.25 ± 1.97	62.5 ± 8.47	16 ± 1.06	16 ± 1.06
	[CoCl_2_(en)_2_]Cl (2)	16 ± 1.97	31.25 ± 1.97	62.5 ± 4.95	16 ± 2.41	16 ± 1.97
	Amphotericin B	–		–	0.5 ± 0.04	64 ± 8.27
*C. krusei* ATCC 6259	[CoCl_2_(dap)_2_]Cl (1)	62.5 ± 8.47	125 ± 16.16	125 ± 8.08	62.5 ± 4.04	125 ± 8.08
	[CoCl_2_(en)_2_]Cl (2)	62.5 ± 4.04	125 ± 8.08	125 ± 9.89	62.5 ± 4.95	125 ± 9.89
	Amphotericin B	–	–	–	2 ± 0.16	64 ± 4.13
*C. krusei* Flu^S^59	[CoCl_2_(dap)_2_]Cl (1)	62.5 ± 4.04	62.5 ± 8.47	62.5 ± 4.04	62.5 ± 4.04	125 ± 8.08
	[CoCl_2_(en)_2_]Cl (2)	62.5 ± 8.47	62.5 ± 4.95	125 ± 9.89	62.5 ± 4.95	125 ± 16.16
	Amphotericin B	–	–	–	2 ± 0.32	>128


*^∗^d, day. Data shown are mean ± SD.*

### Ergosterol Assay

The purpose of the test was to determine if cobalt (III) coordination compounds with diamine derivatives bind to ergosterol of the fungal cell membrane. The obtained results show that MIC values of **(1)** and **(2)** for the tested strains are similar to those in ergosterol-free trials. In the case of amphotericin B, there is a clear increase in the MIC compared with the ergosterol-free trial. For strain *C. albicans* ATCC 10231, 12823 and clinical strains *C. glabrata* 11644, the MIC is 0.5 µg/mL for the ergosterol-free test, and an increase to 64 µg/mL is observed after the addition of ergosterol. For *C. glabrata* ATCC 2001 the MIC is 0.25 µg/mL for the ergosterol-free test and an increase to 32 µg/mL is observed after the addition of ergosterol. MIC values for *C. krusei* ATCC 6259 and Flu^S^59 increased to 64 µg/mL and over 128 µg/mL, respectively (**Table 5**).

Test results indicated that the antifungal activity of Co(III) complexes with diamine chelate ligands is not related to the effect on ergosterol cell membranes of yeasts.

### Cytotoxicity Assay

**FIGURE 4:**
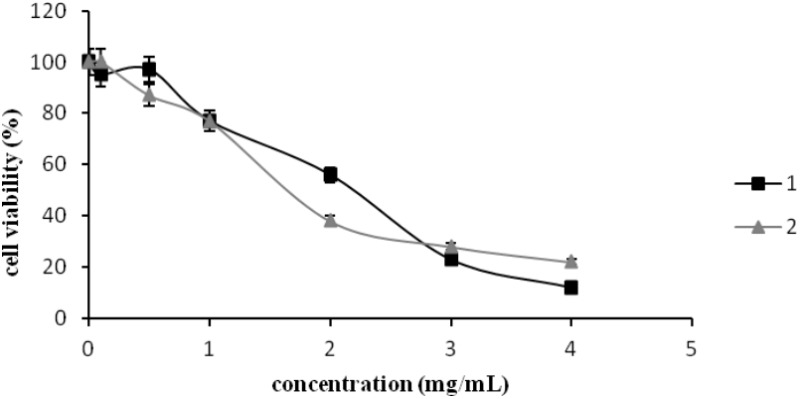
The cytotoxic activity of [CoCl_2_(dap)_2_]Cl (1) and [CoCl_2_(en)_2_]Cl (2) in the concentration range of 0–4000 µg/mL tested against human keratinocyte (HaCaT) cell line measured by MTT assay. Data shown are mean ± SD.

The cytotoxic activity of the compounds was tested on the human keratinocyte (HaCaT) cell line determined with the MTT assay. It was found that in the case of (1) at concentrations up 125 µg/mL, cell viability decreased by 3%, while in case of (2) (in the same concentration range) no growth inhibition was observed (**Figure 4**). Interestingly, at the concentration of 500 µg/mL of **(1)** and **(2)**, cell viability decreased by 3 and 13%, respectively.

### Erythrocyte Lysis Assay

**FIGURE 5:**
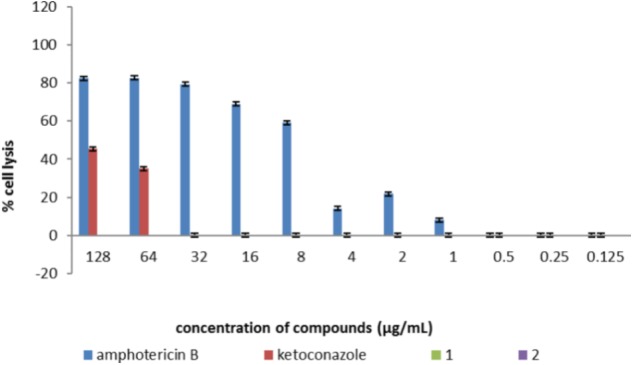
The effect of [CoCl_2_(dap)_2_]Cl (1), [CoCl_2_(en)_2_]Cl (2), and control antifungal compounds (amphotericin B and ketoconazole) on erythrocytes studied in the concentration range of 0.25–128 µg/mL. The results are presented in the form of % hemolysis of red blood cells. Lack of bars determining the percentage of hemolysis by tested compounds means no hemolysis (0% hemolysis of red blood cells). Data shown are mean ± SD.

Erythrocyte lysis assay was performed to study the toxicity of Co(III) complexes on red blood cells. For comparison purposes amphotericin B and ketoconazole were also examined as standard drugs. Analysis were conducted in the concentration range of the tested compounds from 128 to 0.125 µg/mL. Studies with **(2)** showed no hemolysis throughout the whole concentration range. In the case of **(1)**, 0.26–0.11% hemolysis was induced at the concentration range of 128–2 µg/mL, respectively. At concentrations below 2 µg/mL (1–0.125 µg/mL) **(1)** hemolysis was not demonstrated. While amphotericin B at the concentration of 128 µg/mL was highly toxic, showing around 82% cell lysis, hemolysis was not present below 1 µg/mL. However, ketoconazole at the concentration of 128 µg/mL presented about 45.5% hemolysis and at the concentration of 64 µg/mL, 35.1%. At a concentration of 32 µg/mL and lower no hemolysis was observed (**Figure 5**).

### Serial Passages Assay

**FIGURE 6:**
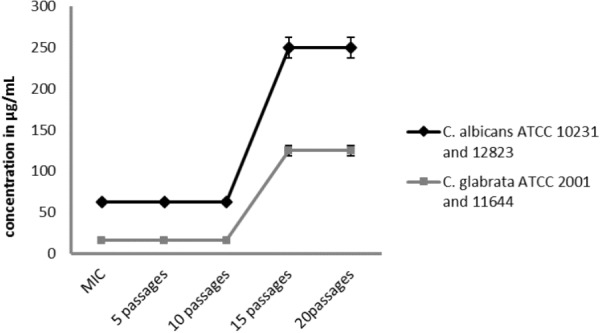
Changes in the initial activity of the compound [CoCl_2_(dap)_2_]Cl (1) and [CoCl_2_(en)_2_]Cl (2) after 20 subsequent passages of reference and clinical strains of *Candida albicans* and *Candida glabrata* in the presence of compounds below their active concentrations (0.5 × MIC). Data shown are mean ± SD.

Four strains of *Candida* spp. were tested by serial passages in a medium supplemented with **(1)** or **(2)** below their active concentrations (0.5 × MIC) in order to evaluate the reduction in susceptibility to the tested compounds (**Table 6** and **Figure 6**). Initial MIC values of compound 1 and 2 and those after each five passages were determined by a broth microdilution method. Serial passaging of *Candida* spp. in the presence of tested compounds caused increasing MIC values. After 15 passages of *C. albicans* and *C. glabrata* strains, their susceptibility was reduced four and eight times, respectively, in comparison to that of the initial MICs. No further reduction in activity was observed after the next five passages.

**Table 6:** MIC (µg/mL) values in the presence of [CoCl_2_(dap)_2_]Cl (1) and [CoCl_2_(en)_2_]Cl (2) passaging results for selected strains of *Candida* spp.

Strains	Compound	Initial MIC (µg/mL)	MIC after 20 passages (µg/mL)
*C. albicans* ATCC 10231	[CoCl_2_(dap)_2_]Cl (1)	62.5 ± 4.04	250 ± 19.79
	[CoCl_2_(en)_2_]Cl (2)	62.5 ± 8.47	250 ± 16.16
*C. albicans* 12823	[CoCl_2_(dap)_2_]Cl (1)	62.5 ± 4.95	250 ± 16.16
	[CoCl_2_(en)_2_]Cl (2)	62.5 ± 4.04	250 ± 32.31
*C. glabrata* ATCC 2001	[CoCl_2_(dap)_2_]Cl (1)	16 ± 1.97	125 ± 8.08
	[CoCl_2_(en)_2_]Cl (2)	16 ± 1.06	125 ± 9.89
*C. glabrata* 11644	[CoCl_2_(dap)_2_]Cl (1)	16 ± 1.06	125 ± 9.89
	[CoCl_2_(en)_2_]Cl (2)	16 ± 2.41	125 ± 8.08


*Data shown are mean ± SD.*

### Stability Assay

The stability of compounds **(1)** and **(2)** in solution over time was evaluated. The MIC and MFC values of the Co(III) complexes against two reference strains of *Candida* spp. were determined after 14 and 30 days of incubation at the temperature of 25–28°C under dark and light conditions. There was no decrease in activity of the compounds after the investigated incubation period. Also the light conditions during the stability test did not affect their activity (Supplementary Tables S1, S2).

## Discussion

In this study the [CoCl_2_(en)_2_]Cl and [CoCl_2_(dap)_2_]Cl, Co(III) complexes with diamine chelate ligands, were tested against a wide range of *Candida* spp. strains. The antiviral, antibacterial, antitumor and antifungal activities of Co(II) and Co(III) coordination compounds has been widely described (Arali et al., 1993; Yadave and Patil, 1997; Walker et al., 2003; Yilmaz and Cukurovali, 2003; Liang et al., 2004; Belicchi-Ferrari et al., 2005; Lv et al., 2006; Zhong et al., 2006; Bisceglie et al., 2007; Penumaka and Satyanarayana, 2007; Konidaris et al., 2010). However, only Co(III) coordination compounds remain the center of researchers’ interest. Previous studies have reported high antimicrobial activity of optical isomer Co(III) complexes of N,N-(di)donor ligand ethylenediamine [Co(en)_3_](NO_3_)_3_ with other metal ion complexes. The coordination compounds of cobalt (II)-pyridine-amide bidentate and tridentate ligands has also been studied (Petrusewicz et al., 1999), and showed strong anti-bacterial properties toward *Pseudomonas*, *Escherichia coli*, *Shigella flexneri*, and *Klebsiella planticola.* There is a large group of cobalt coordination compounds (III) which also exhibit potent antiviral properties, and are obtained by oxidation of their precursors of cobalt as the second center oxidation. An example would be CTC-96, which is a potent agent against herpes simplex type 1 (HSV-1) (Ahn et al., 1998). This compound is applied in medicines that prevent conjunctivitis and also acts against the epidermal inflammation of the cornea. The cobalt complexes with organic ligands are very interesting as potential candidates as antimicrobial agents due to their therapeutical uses as antiviral, antibacterial, antitumor and antifungal, antiparasitic, transferrin transport, anti-inflammatory agents. A. Mishura found that coordination compounds of Co(III) with pyridine-amide as bidentate and tridentate ligands exhibited significant antibacterial activity against the resistant strains of *Pseudomonas*, *E. coli*, and reference strains of *Shigella* (Mishra et al., 2008). Kumar and Arunachalam (2008) reported that surfactant-Co(III) complexes showed good antibacterial and antifungal activity. Nagababu et al. (2008) screened a large number of bis(ethylenediamine)cobalt(III) cations against bacteria (*E. coli, S. typhimurium, P. aeruginosa, P. vulgaris, S. aureus, and E. faecalis*) and fungi (*A. niger, N. crassa, and F. oxysporum*). They showed that some of these compounds had higher antibacterial activity than the tested antibiotics (gentamicin, streptomycin, penicillin, chloramphenicol, or ciprofloxacin). The studied complexes exhibited also good antifungal activity (Nagababu et al., 2008).

In the present study Co(III) complexes with diamine chelate ligands exhibited strong antifungal activity against *Candida* spp. A previous report using control microbial strains, like Gram-positive bacteria *Enterococcus hirae* ATCC 10541, *Staphylococcus aureus* ATCC 6538, Gram-negative bacteria *Escherichia coli* ATCC 8739, *Proteus vulgaris* 4635, *Pseudomonas aeruginosa* ATCC 9077 and yeast *C. albicans* ATCC 10231 demonstrated antibacterial and antifungal activity of these compounds. However, the strongest effect was observed against *C. albicans*. Moreover, the tested complexes of Co(III) showed approximately 4–10 fold greater activity than the ligands alone (Chylewska et al., 2013). Our work suggests that the tested compounds have potential as antifungal agents due to their low MIC values. The MIC_S_ of **(1)** and **(2)** ranged from 16 to 125 µg/mL. Among the tested *Candida* strains the most sensitive proved to be *C. glabrata* (MIC was 16 µg/mL). The explanation as to why *C. glabrata* is much more sensitive toward these kinds of compounds seems to be that *C. glabrata* is not polymorphic, it has no ability to form hyphae or pseudohyphae and grows only as blastoconidia (Silva et al., 2012) in contrast to *C. albicans* or *C. krusei*. Indeed, in the microscope slide of the *C. albicans* culture we found characteristic structures – blastoconidia or pseudohyphae, absent in *C. glabrata*.

To study the reduction in susceptibility to the diamine complexes with Co(III) analogs, the serial passages assay was performed. The effectiveness of the tested compounds decreased during the multiple passages in the medium supplemented with the subinhibitory concentrations of the compounds. No differences in induction of fungal resistance between tested compounds were observed, while for reference and clinical strains of *C. glabrata* the MIC value increased two times compared to *C. albicans* strains. The study included in the FDA elaboration showed that MIC values for the chosen clinical strain of *C. albicans* for fluconazole after 15 passages increased from 0.12 to 2 µg/mL (about 16 times)^1^ (FDA, 2009). However, Kumar R. and Shukla P. K. conducted serial passaging of a reference strain of *C. albicans* in medium supplemented with amphotericin B below its active concentrations. After 60 passages, a huge increase in MIC values was observed (MIC increased from 0.06 to 128 µg/mL), over 2000 times (Kumar and Shukla, 2010). It is well-known that resistance appears in microorganisms due to the selective pressure caused by the use of antimicrobial compounds. Sensitive microorganisms become resistant as a result of changes in their genome. Fungi, like most human pathogens, are undergoing evolutionary changes, and their adaptive abilities lead to the development of drug resistance. The occurrence of antifungal drug resistance among fungi is associated with the constantly increasing consumption of these kinds of compounds in therapy and increasing concentrations in the environment as an effect of human activity in agriculture, food industry as well as forestry. The first reports on the resistance of *Candida* species to azoles (ketoconazole) appeared in the eighties, shortly after the onset of the AIDS epidemic. It is known that people with AIDS are chronically consuming antifungal drugs (fluconazole) (Romanowska, 2008).

Simultaneously with the serial passages assay, the stability of compounds was examined over a period of 30 days. We did not observed a decrease in activity of the compounds, indicating no disintegration of compounds over time.

Due to its anti-*Candida* activity, Co(III) complexes with diamine chelate ligands were then studied using optical and transmission electron microscope. In optical microscope analysis the *Candida* cells showed altered morphology in the presence of **(1)** or **(2)** while the untreated cells were seen intact. Moreover, a tendency for cell aggregation was observed, similarly to the commercial antifungal drug – amphotericin B. Furthermore, trypan blue assay showed blue-stained dead yeast cells, which may suggest change of membrane permeability characteristic for dead cells. Using TEM we observed strong anti-fungal activity of tested complexes of Co(III) that led to changes in yeast cell morphology, indicating significant disruptions in the molecular machinery of cells.

To study the action of **(1)** or **(2)** on the fungal cell wall we performed an assay with sorbitol. Sorbitol is an osmotic protector stabilizing fungal protoplasts. Cells protected by this sugar alcohol can grow in the presence of antimicrobial compounds acting on fungal cell wall synthesis, whereas growth would be inhibited in the absence of sorbitol. The increase of MIC in the experiments with sorbitol could indicate the cell wall as a potential target of action of the compound. The sorbitol added to the sample causes osmotic stress to the cell. Then perhaps more of the compound penetrates the cell. Similar MIC values for samples with and without sorbitol indicate no effect of the tested compound on the fungal cell wall synthesis.

To examine the possible antifungal effect of Co(III) complexes with diamine chelate ligands on the fungal cell membrane, the ability of the tested compounds to form complexes with ergosterol was also investigated. Ergosterol is a sterol, comprising part of the fungal cell membrane. This compound does not occur in human or animals cells, so it is the target of most antifungal agents. The purpose of the test was to determine if cobalt (III) coordination compounds with diamine derivatives bind to ergosterol of the fungal cell membrane. If the tested compound interacts with ergosterol of the fungal cell membrane, an increase in the MIC value can be observed in the ergosterol assay. This is due to the binding of the analyzed substance to the free ergosterol, so that a higher concentration of the compound is needed to inhibit fungal growth. The above-mentioned mechanism of action is displayed by amphotericin B, used in the control studies. This antifungal binds to the membrane of the fungal cell membrane (mainly ergosterol), and reorients the sterol molecules, which results in damage to the cytoplasmic membrane. Potassium ions, larger diameter ions, inert particles and macromolecules flow sequentially from the cell. There is a disturbance in metabolism and cell death (Andriole, 1999). The ergosterol binding assay showed that MICs of the tested Co(III) complexes had similar values in probes with and without additional ergosterol, whereas control trials containing amphotericin B were characterized by a significant increase in MIC values (for example, for *C. albicans* and *C. glabrata* strains MICs increased from 0.5 to 64 µg/mL). The large increase in the MIC of the antifungal agent implies its mechanism of action (binding of amphotericin B to extracellular ergosterol causes less drug molecules to bind to ergosterol of the cell membrane). Similar analysis using the ergosterol assay have been reported and employ this method in the investigation of the mechanism of action of compounds (Sharma et al., 2016). Thus no increase in MIC values for tests with and without ergosterol indicates that the mechanism of action of **(1)** or **(2)** complexes does not affect the interaction with ergosterol cell membrane.

In our study the synergististic activity of **(1)** and **(2)** with amphotericin B and ketoconazole was also tested. The Co(III) complexes showed synergistic activity with ketoconazole against *C. albicans* strains, both reference (ATCC 10231) and clinical strains (12823) (FIC 0.51 and 0.26, respectively). The same effect was observed in the case of *C. krusei* ATCC 6259, *C. krusei* Flu^S^59, *C. krusei* Flu^R^176 and *C. tropicalis* but only for **(1)** (FIC 0.26, 0.29 and 0.50, 0.51, respectively). The tested compounds increased the antifungal activity of ketoconazole against several strains of *Candida* species (*C. albicans, C. krusei*, and *C. tropicalis*). It has been already shown that the tested Co(III) complexes with diamine chelate ligands alone induce ultrastructural changes in *Candida* strains, indicating significant disorganization in the molecular machinery of fungal cells. Ketoconazole belongs to azoles. The mechanism of action of this class of drugs is to block the cytochrome P450-dependent 14-α-demethylase activity, thereby inhibiting ergosterol synthesis and finally damaging the cell membrane of the fungus. The cytochrome P450 are the transmembrane proteins associated with the membrane of the endoplasmic reticulum and the inner mitochondrial membrane. We hypothesize that increased antifungal activity of ketoconazole in the presence of the Co(III) complexes may be due to damage of the mitochondrial membrane or the membrane of the endoplasmic reticulum. Indeed, damages to the mitochondria and endoplasmic reticulum membranes of the tested *Candida* strains were observed by Transmission Electron Microscopy (**Figures 3B3,C3**). Moreover, it is worth noting that the tested compounds contain N-donor ligands in their composition. Tümer et al. (1999) has suggested that the ligands with the N or O donor system might inhibit the synthesis of enzymes, which require a free hydroxyl group for their activity. These kinds of enzymes are especially susceptible to the deactivation by the ion of the complexes (Tümer et al., 1999). In turn, El-Ayaan and Abdel-Aziz (2005) have described a cobalt(III) complex with the rigid bidentate nitrogen ligand *bis*[*N*-(2,6-diisopropylphenyl)imino] acenaphthene (Pr-BIAN) and its potent antibacterial activity. The researchers suggested that the high antibacterial activity was associated with bulkiness of the *N*-bisimine derivatives which decreases the polarity of the molecule due to the chelation of the cation. It causes an increase in the lipophilicity of the complex and thus enabling better penetration through the cellular membrane and blocking the metal binding sites in enzymes (El-Ayaan and Abdel-Aziz, 2005). Another mode of action of Co(III) complexes with surfactants based on binding to DNA was also proposed (Kumar and Arunachalam, 2008). However, further research is needed to understand the exact mechanism of action.

*In vitro* haemolytic activity of Co(III) complexes and two commercially used antifungal drugs (amphotericin B and ketoconazole) was studied to estimate the toxic effects of these compounds on red blood cells. The tested complexes **(1)** and **(2)** were found to be non-toxic throughout the whole concentration range, containing MIC values, while amphotericin B in MIC concentrations showed toxicity of about 22% for 2 µg/mL, 8% for 1 µg/mL. At the concentration below 1 µg/mL, the compound was not hemolytic. Compounds were also checked for their toxicity against the human keratinocyte (HaCaT) cell line. No cytotoxic activity was detected in the MIC range.

The obtained results indicate that Co(III) coordination complexes with diamine chelate ligands could be developed into a potential antifungal drug. They show excellent antifungal activity against a broad spectrum of *Candida* species and are non-toxic in the concentrations tested for antimicrobial activity. However, further and wider spectrum of tests are necessary to understand their mode of action.

## Author Contributions

KT designed the research, performed the experiments, analyzed the data, and wrote the manuscript. AC designed and synthesized the Co(III) complexes with diamine chelate ligands. AK performed the cytotoxicity assay and reviewed the manuscript. KW contributed in design of experiments and critically reviewed the manuscript. Also, KT, AK, and KW contributed in the revision of manuscript.

## Conflict of Interest Statement

The authors declare that the research was conducted in the absence of any commercial or financial relationships that could be construed as a potential conflict of interest.

## Footnotes

1 https://www.drugs.com

## References

[B1] Achkar J. M. Fries B. C. (2010). Candida infections of the genitourinary tract. *Clin. Microbiol. Rev.* 23 253–273. 10.1128/CMR.00076-09 20375352 PMC2863365

[B2] Ahn S. H. Seo D. W. Ko Y. K. Sung D. S. Bae G. U. Yoon J. W. (1998). NO/cGMP pathway is involved in exocrine secretion from rat pancreatic acinar cells. *Arch. Pharm. Res.* 21 657–663. 10.1007/BF02976753 9868533

[B3] Andriole V. T. (1999). Current and future antifungal therapy: new targets for antifungal therapy. *J. Antimicrob. Chemother.* 44 152–162. 10.1093/jac/44.2.151 10473222

[B4] Arali V. H. Revankar V. K. Mahale V. B. (1993). Synthesis, characterization and biological studies of 2-(3,5-dimethylpyrazol-1-yl)benzothiazole complexes of cobalt(II), nickel(II) and cooper(II). *Transit. Met. Chem.* 18 158–162. 10.1007/BF00139947

[B5] Banasiuk R. Frackowiak J. E. Krychowiak M. Matuszewska M. Kawiak A. Ziabka M. (2016). Synthesis of antimicrobial silver nanoparticles through a photomediated reaction in an aqueous environment. *Inter. J. Nanomed.* 11 315–324. 10.2147/IJN.S93611 26855570 PMC4725629

[B6] Belicchi-Ferrari M. Bisceglie F. Casoli C. Durot S. Morgerstern-Badarau I. Pelosi G. (2005). Copper(II) and cobalt(III) pyridoxal thiosemicarbazone complexes with nitroprusside as counterion: syntheses, electronic properties, and antileukemic activity. *J. Med. Chem.* 48 1671–1679. 10.1021/jm049529n 15743209

[B7] Bisceglie F. Baldini M. Belicchi-Ferrari M. Buluggiu E. Careri M. Pelosi G. (2007). Metal complexes of retinoid derivatives with antiproliferative activity: synthesis, characterization and DNA interaction studies. *Eur. J. Med. Chem.* 42 627–634. 10.1016/j.ejmech.2006.12.019 17296250

[B8] Brown J. M. (1999). The hypoxic cell: a target for selective cancer therapy – Eighteenth Bruce F. Cain Memorial Award Lecture. *Cancer Res.* 59 5863–5870. 10606224

[B9] Chylewska A. Turecka K. Dąbrowska A. Werel W. Chmurzyński L. (2013). Synthesis, physicochemical characterization and antimicrobial activity of Co (III) complexes with diamine chelate ligands. *IJAPBC* 2 454–464.

[B10] Clinical and Laboratory Standards Institute [CLSI] (2008). *Method M27-A3 “Reference Method for Dilution Antifungal Susceptibility Testing of Yeast; Approved Standard*, 3rd Edn Wayne, PA: CLSI.

[B11] Dachs G. U. Tozer G. M. (2000). Hypoxia modulated gene expression: angiogenesis, metastasis and therapeutic exploitation. *Eur. J. Cancer* 36 1649–1660. 10.1016/S0959-8049(00)00159-3 10959051

[B12] El-Ayaan U. Abdel-Aziz A. A. M. (2005). Synthesis, antimicrobial activity and molecular modeling of cobalt and nickel complexes containing the bulky ligand: bis[N-(2,6-diisopropylphenyl)imino]acenaphthene. *Eur. J. Med. Chem.* 40 1214–1221. 10.3390/ph3061711 16126307

[B13] Espinel-Ingroff A. Colombo A. L. Cordoba S. Dufresne P. J. Fuller J. Ghannoum M. (2016). International evaluation of MIC distributions and epidemiological cutoff value (ECV) definitions for *Fusarium* species identified by molecular methods for the CLSI broth microdilution method. *Antimicrob. Agents Chemother.* 60 1079–1084. 10.1128/AAC.02456-15 26643334 PMC4750715

[B14] FDA (2009). *Professionals PI, Amphotericin B - FDA Prescribing Information, Side Effects and Uses*. Available at: https://www.drugs.com

[B15] Groll A. H. Piscitelli S. C. Walsh T. J. (1998). Clinical pharmacology of systemic antifungal agents: a comprehensive review of agents in clinical use, current investigational compounds, and putative targets for antifungal drug development. *Adv. Pharmacol.* 44 343–500. 10.1016/S1054-3589(08)60129-5 9547888

[B16] Hałasa R. Turecka K. Orlewska C. Werel W. (2014). Comparison of fluorescence optical respirometry and microbroth dilution methods for testing antimicrobial compounds. *J. Microbiol. Methods* 107 98–105. 10.1016/j.mimet.2014.09.008 25307692

[B17] Hof H. (2006). A new, broad-spectrum azole antifungal: posaconazole - mechanisms of action and resistance, spectrum of activity. *Mycoses* 49 2–6. 10.1111/j.1439-0507.2006.01295.x 16961575

[B18] Jackson B. E. Wilhelmus K. R. Mitchell B. M. (2007). “Genetically regulated filamentation contributes to *Candida albicans* virulence during corneal infection. *Microb. Pathog.* 42 88–93. 10.1016/j.micpath.2006.11.005 17241762 PMC1892154

[B19] Jastrzębowska K. Gabriel I. (2015). Inhibitors of amino acids biosynthesis as antifungal agents. *Amino Acids* 47 227–249. 10.1007/s00726-014-1873-1 25408465 PMC4302243

[B20] Konidaris K. F. Raptopoulou C. P. Psycharis V. Perlepes S. P. Manessi-Zoupa E. Stamatatos T. C. (2010). Use of the 2-Pyridinealdoxime/N,N’-Donor ligand combination in cobalt (III) chemistry: synthesis and characterization of two cationic mononuclear cobalt (III) complexes. *Bioinorg. Chem. Appl.* 7 1–7. 10.1155/2010/159656 20721276 PMC2913532

[B21] Kontoyiannis D. P. Mantadakis E. Samonis G. (2003). Systemic mycoses in the immunocompromised host: an update in antifungal therapy. *J. Hosp. Infect.* 53 243–258. 10.1053/jhin.2002.1278 12660121

[B22] Kuhn D. M. Ghannoum M. A. (2004). *Candida* biofilms: antifungal resistance and emerging therapeutic options. *Curr. Opin. Investig. Drugs* 5 186–197. 15043393

[B23] Kumar R. Shukla P. K. (2010). Amphotericin B resistance leads to enhanced proteinase and phospholipase activity and reduced germ tube formation in *Candida albicans*. *Fungal Biol.* 114 189–197. 10.1016/j.funbio.2009.12.003 20943129

[B24] Kumar R. S. Arunachalam S. (2008). Synthesis, micellar properties, DNA binding and antimicrobial studies of some surfactant-cobalt(III) complexes. *Biophys. Chem.* 136 136–144. 10.1016/j.bpc.2008.05.007 18571829

[B25] Leite M. C. A. Bezerra A. P. B. Sousa J. P. Guerra F. Q. S. Lima E. O. (2014). Evaluation of antifungal activity and mechanism of action of citral against *Candida albicans*. *J. Evid. Based Complementary Altern. Med.* 11 1–9. 10.1155/2014/378280 25250053 PMC4163309

[B26] Liang F. Wang P. Zhou X. Li T. Li Z. Lin H. (2004). Nickel(II) and cobalt(II) complexes of hydroxyl-substituted triazamacrocyclic ligand as potential antitumor agents. *Bioorg. Med. Chem. Lett.* 14 1901–1904. 10.1016/j.bmcl.2004.01.089 15050623

[B27] Lv J. Liu T. Cai S. Wang X. Liu L. Wang Y. (2006). Synthesis, structure and biological activity of cobalt(II) and copper(II) complexes of valine-derived schiff bases. *J. Inorg. Biochem.* 100 1888–1896. 10.1016/j.jinorgbio.2006.07.014 16965817

[B28] MacCallum D. M. (2012). Hosting infection: experimental models to assay *Candida* virulence. *Int. J. Microbiol.* 2012:363764. 10.1155/2012/363764 22235206 PMC3253448

[B29] Mahmound A. G. Louis B. R. (1999). Antifungal agents: mode of action, mechanisms of resistance, and correlation of these mechanisms with bacterial resistance. *Clin. Microbiol. Rev.* 12 501–517. 10515900 10.1128/cmr.12.4.501 PMC88922

[B30] Meletiandis J. Mouton J. W. te Dorsthorst D. T. A. Verweij P. E. (2005). Assesing in vitro combination of antifungal drugs against yeasts and filamentous fungi: comparison of different drug interaction models. *Med. Mycol.* 43 133–152. 10.1080/13693780410001731547 15832557

[B31] Mikulska M. del Bono V. Ratto S. Viscoli C. (2012). Occurrence, presentation and treatment of candidemia. *Expert Rev. Clin. Immunol.* 8 755–765. 10.1586/eci.12.52 23167687

[B32] Mishra A. Kaushik N. K. Verma A. K. Gupta R. (2008). Synthesis, characterization and antibacterial activity of Co (III) complexes with pyridine-amide ligands. *Eur. J. Med. Chem.* 43 2189–2196. 10.1016/j.ejmech.2007.08.015 17959275

[B33] Morrell M. Fraser V. J. Kollef M. H. (2005). Delaying the empiric treatment of *Candida* bloodstream infection until positive blood culture results are obtained: a potential risk factor for hospital mortality. *Antimicrob. Agents Chemother.* 49 3640–3645. 10.1128/AAC.49.9.3640-3645.2005 16127033 PMC1195428

[B34] Mossman T. (1983). Rapid colorimetric assay for cellular growth and survivals: application to proliferation and cytotoxicity assays. *J. Immunol. Methods* 65 55–63. 10.1016/0022-1759(83)90303-4 6606682

[B35] Nagababu P. Latha J. N. L. Pallavi P. Harish S. Satyanarayana S. (2008). Studies on antimicrobial activity of cobalt(III) ethylenediamine complexes. *Can. J. Microbiol.* 52 1247–1254. 10.1139/w06-087 17473894

[B36] Naglik J. R. Moyes D. L. Wächtler B. Hube B. (2011). *Candida albicans* interactions with epithelial cells and mucosal immunity. *Microbes Infect.* 13 963–976. 10.1016/j.micinf.2011.06.009 21801848 PMC3185145

[B37] Ngo H. X. Garneau-Tsodikova S. Green K. D. (2016). A complex game of hide and seek: the search for new antifungals. *Med. Chem. Commun.* 7 1285–1306. 10.1039/C6MD00222F 27766140 PMC5067021

[B38] Odds F. C. (2003a). Fluconazole plus amphotericin B combinations are not contraindicated and may add benefit for the treatment of candidemia. *Clin. Infect. Dis.* 36 1229–1231. 10.1086/374856 12746766

[B39] Odds F. C. (2003b). Synergy, antagonism, and what the chequerboard puts between them. *J. Antimicrob. Chemother.* 52:1. 10.1093/jac/dkg301 12805255

[B40] Ostrosky-Zeichner L. Casadevall A. Galgiani J. N. Odds F. C. Rex J. H. (2010). An insight into the antifungal pipeline: selected new molecules and beyond. *Nat. Rev. Drug Discov.* 9 719–727. 10.1038/nrd3074 20725094

[B41] Penumaka N. Satyanarayana S. (2007). DNA binding and photocleavage studies of cobalt(III) polypyridine complexes: [Co(en)2PIP]3+, [Co(en)2IP]3+, and [Co(en)2phen-dione]3+. *Bioinorg. Chem. Appl.* 8 1–8. 10.1155/2007/54562 18253471 PMC1997276

[B42] Perea S. Lopez- Ribot J. L. Kirkpatrick W. R. McAtee R. K. Santillan R. A. Martinez M. (2001). Prevalence of molecular mechanisms of resistance to azole antifungal agents in *Candida albicans* strains displaying high-level fluconazole resistance isolated from human immunodeficiency virus-infected patients. *Antimicrob. Agents Chemother.* 45 2676–2684. 10.1128/AAC.45.10.2676-2684.2001 11557454 PMC90716

[B43] Pereira J. V. Freires I. A. Castilho A. R. da Cunha M. G. Alves H. D. Rosalen P. I. (2016). Antifungal potential of *Sideroxylon obtusifolium* and *Syzygium cumini* and their mode of action against *Candida albicans*. *Pharm. Biol.* 54 2312–2319. 10.3109/13880209.2016.1155629 26987037

[B44] Petrusewicz J. Siluk D. Kaliszan R. Foks H. Nowakowska E. (1999). Comparative study of antithrombotic and antiaggregatory activity of acetylsalicylic acid, ticlopidine and a new noncarboxylic acid antiinflammatory pyrazine derivative HF90. *Acta Pol. Pharm.* 56 463–467. 10715891

[B45] Pfaller M. A. Diekema D. J. (2004). Rare and emerging opportunistic fungal pathogens: concern for resistance beyond *Candida albicans* and *Aspergillus fumigatus*. *J. Clin. Microbiol.* 42 4419–4431. 10.1128/JCM.42.10.4419-4431.2004 15472288 PMC522363

[B46] Pfaller M. A. Diekema D. J. (2007). Epidemiology of invasive candidiasis: a persistent public health problem. *Clin. Microbiol. Rev.* 29 133–163. 10.1128/CMR.00029-06 17223626 PMC1797637

[B47] Pierce C. G. Srinivasan A. Uppuluri P. Ramasubramanian A. K. Lopez-Ribot J. L. (2013). Antifungal therapy with an emphasis on biofilms. *Curr. Opin. Pharmacol.* 13 726–730. 10.1517/17460441.2013.807245 24011516 PMC3795934

[B48] Ramage G. Vande Walle K. Wickes B. L. Lopez-Ribot J. L. (2001). Standardized metod for in vitro antifungal susceptibility testing of *Candida albicans* biofilms. *Antimicrob. Agents Chemother.* 45 2475–2479. 10.1128/AAC.45.9.2475-2479.2001 11502517 PMC90680

[B49] Roemer T. Krysan D. J. (2014). Antifungal drug development: challenges, unmet clinical needs, and new approaches. *Cold Spring Harb. Perspect. Med.* 4:a019703. 10.1101/cshperspect.a01973 24789878 PMC3996373

[B50] Romanowska E. (2008). “Fungal resistance mechanisms on antifungal agents,” in *Hospital Infections*, ed. Dzerżanowska D. (Bielsko-Biala: α-Medica press), 497–507.

[B51] Rosenbach A. Dignard D. Pierce J. V. Whiteway M. Kumamoto C. A. (2010). Adaptations of *Candida albicans* for growth in the mammalian intestinal tract. *Eukaryot. Cell* 9 1075–1086. 10.1128/EC.00034-10 20435697 PMC2901676

[B52] Sanglard D. Odds F. C. (2002). Resistance of *Candida* species to antifungal agents: molecular mechanisms and clinical consequences. *Lancet Infect. Dis.* 2 73–85. 10.1016/S1473-3099(02)00181-0 11901654

[B53] Scorzoni L. de Paula e Silva A. C. A. Marcos C. M. Assato P. A. de Melo W. C. M. A. de Oliveira H. C. (2017). Antifungal therapy: new advances in the under standing and treatment of mycosis. *Front. Microbiol.* 8:36 10.3389/fmicb.2017.00036 PMC5253656 28167935

[B54] Sharma Y. Khan L. A. Manzoor N. (2016). Anti-*Candida* activity of geraniol involves disruption of cell membrane integrity and function. *J. Mycol. Med.* 26 244–254. 10.1016/j.mycmed.2016.04.004 27554866

[B55] Silva S. Negri M. Henriques M. Oliveira R. Williams D. W. Azeredo J. (2012). *Candida glabrata*, *Candida parapsilosis* and *Candida tropicalis*: biology, epidemiology, pathogenicity, and antifungal resistance. *FEMS* 36 288–305. 10.1111/j.1574-6976.2011.00278.x 21569057

[B56] Sundriyal S. Sharma R. K. Jain R. (2006). Current advances in antifungal targets and drug development. *Curr. Med. Chem.* 13 1321–1335. 10.2174/092986706776873023 16712473

[B57] Sydnor E. R. M. Perl T. M. (2011). Hospital epidemiology and infection control in acute-care settings. *Clin. Microbiol. Rev.* 24 141–173. 10.1128/CMR.00027-10 21233510 PMC3021207

[B58] Sykłowska-Baranek K. Pietrosiuk A. Naliwajski M. R. Kawiak A. Jeziorek M. Wyderska S. (2012). Effect of L-phenylalanine on PAL activity and production of naphthoquinone pigments in suspension cultures of *Arnebia euchroma* (Royle) Johnst. *In Vitro Cell Dev. Biol. Plant* 48 555–564. 10.1007/s11627-012-9443-2 23049233 PMC3462983

[B59] Tümer M. Köksal H. Sener M. K. Serin S. (1999). Antimicrobial activity studies of the binuclear metal complexes derived from tridentate Schiff base ligands. *Transit. Met. Chem.* 24 414–420. 10.1023/A:1006973823926 22580137

[B60] Walker G. W. Gene R. J. Sargeson A. M. Behm C. A. (2003). Surface-active cobalt cage complexes: synthesis, surface chemistry, biological activity, and redox properties. *Dalton Trans.* 15 2992–3001. 10.1039/B302230G

[B61] Wu T. Mitchell B. Carothers T. Coats D. Brady-McCreery K. Paysse E. (2003). Molecular analysis of the pediatric ocular surface for fungi. *Curr. Eye Res.* 26 33–36. 10.1076/ceyr.26.1.33.14253 12789534

[B62] Yadave M. S. Patil S. A. (1997). Synthesis, characterization and biological studies of cobalt(II) and nickel(II) complexes with new Schiff bases. *Transit. Met. Chem.* 22 220–224. 10.1023/A:1018400121316

[B63] Yilmaz I. Cukurovali A. (2003). Synthesis, characterization and antimicrobial activity of the Schiff bases derived from 2,4-disubstituted thiazole and 3-methoxy salicylaldehyde and their cobalt(II), copper(II), nickel(II) and zinc(II) complexes. *Transit. Met. Chem.* 28 399–404. 10.1023/A:1023630209043

[B64] Zaoutis T. E. Argon J. Chu J. Berlin J. A. Walsh T. J. Feudtner C. (2005). The epidemiology and attributable outcomes of candidemia in adults and children hospitalized in the United States: a propensity analysis. *Clin. Infect. Dis.* 41 1232–1239. 10.1086/496922 16206095

[B65] Zhong X. Yi J. Sun J. Wei H. L. Liu W. S. Yu K. B. (2006). Synthesis and crystal structure of some transition metal complexes with a novel bis-Schiff base ligand and their antitumor activities. *Eur. J. Med. Chem.* 41 1090–1092. 10.1016/j.ejmech.2006.05.009 16782235

