# Supplementary material for: Antifungal Activity and Mechanism of Action of the Co(III) Coordination Complexes With Diamine Chelate Ligands Against Reference and Clinical Strains of Candida spp
Source: Front Microbiol. 2018 Jul 18;9:1594. doi: 10.3389/fmicb.2018.01594 (PMC6058090; doi:10.3389/fmicb.2018.01594)
Supplement: Supplementary file 2 [file Table_2.pdf]

Supplementary Material

Antifungal activity and mechanism of action of the Co(III) coordination complexes with diamine chelate ligands against reference and clinical strains of *Candida spp*.

Katarzyna Turecka[1*], Agnieszka Chylewska[2], Anna Kawiak[3], Krzysztof Waleron[1]

*** Correspondence:** Katarzyna Turecka: tureckak@gumed.edu.pl

Supplementary Tables

TABLE S2. MFC values in µg/mL of $[CoCl_2(dap)_2]Cl$ **(1)**, $[CoCl_2(en)_2]Cl$ **(2)** stored in darkness and light against *Candida* strains evaluated after 14 and 30 days. Data shown are mean ± SD.

Strain	Compound	Darkness		Light	
		14 d*	30 d*	14 d*	30 d*
C. albicans ATCC 10231	$[CoCl_2(dap)_2]Cl$ (1)	125±9.19	125±6.94	125±10.41	125±6.94
	$[CoCl_2(en)_2]Cl$ (2)	125±9.89	125±9.19	125±6.94	125±9.89
C. glabrata ATCC 2001	$[CoCl_2(dap)_2]Cl$ (1)	62.5±5.21	62.5±4.04	62.5±4.95	62.5±4.04
	$[CoCl_2(en)_2]Cl$ (2)	62.5±4.95	62.5±4.95	62.5±5.21	62.5±8.47

**d – day*
